# Influenza A and Respiratory Syncytial Virus Trigger a Cellular Response That Blocks Severe Acute Respiratory Syndrome Virus 2 Infection in the Respiratory Tract

**DOI:** 10.1093/infdis/jiac494

**Published:** 2022-12-23

**Authors:** Kieran Dee, Verena Schultz, Joanne Haney, Laura A Bissett, Callum Magill, Pablo R Murcia

**Affiliations:** Medical Research Council-University of Glasgow Centre for Virus Research, Glasgow, United Kingdom

**Keywords:** human airway epithelium, influenza A virus, respiratory syncytial virus, severe acute respiratory syndrome virus 2, virus coinfection

## Abstract

**Background:**

Multiple viruses cocirculate and contribute to the burden of respiratory disease. Virus-virus interactions can decrease susceptibility to infection and this interference can have an epidemiological impact. As humans are normally exposed to a community of cocirculating respiratory viruses, experimental coinfection studies are necessary to understand the disease mechanisms of multipathogen systems. We aimed to characterize interactions within the respiratory tract between severe acute respiratory syndrome virus 2 (SARS-CoV-2) and 2 major respiratory viruses: influenza A virus (IAV), and respiratory syncytial virus (RSV).

**Methods:**

We performed single infections and coinfections with SARS-CoV-2 combined with IAV or RSV in cultures of human bronchial epithelial cells. We combined microscopy with quantification of viral replication in the presence or absence of an innate immune inhibitor to determine changes in virus-induced pathology, virus spread, and virus replication.

**Results:**

SARS-CoV-2 replication is inhibited by both IAV and RSV. This inhibition is dependent on a functional antiviral response and the level of inhibition is proportional to the timing of secondary viral infection.

**Conclusions:**

Infections with other respiratory viruses might provide transient resistance to SARS-CoV-2. It would therefore be expected that the incidence of coronavirus disease 2019 (COVID-19) may decrease during periods of high circulation of IAV and RSV.

## Teaser

Virus-virus interactions impact the infection dynamics of respiratory viruses at multiple levels, from cells to populations. Using 3-dimensional cultures of airway epithelium, we showed that SARS-CoV-2 replication is impaired in coinfections with either influenza A or respiratory syncytial virus.

Respiratory viral infections are caused by a diverse group of viruses, including influenza A and B viruses (IAV and IBV), severe acute respiratory syndrome virus 2 (SARS-CoV-2), respiratory syncytial virus (RSV), and human rhinovirus (HRV). Recent studies showed the occurrence of virus-virus interactions at various scales, from populations to cells. Analyses of diagnostic data in a large population showed statistical evidence of positive and negative interactions among respiratory viruses at the epidemiological level, and mathematical simulations suggested that negative interactions could be mediated by transient innate immune responses [1]. Clinical and experimental studies using air-liquid interface (ALI) cultures of respiratory epithelium support this hypothesis as they showed that some viruses trigger an interferon (IFN)-mediated response that can block a secondary viral infection [2–4]. Experimental coinfections using animal models supported the occurrence of virus-virus interactions in vivo [5–8].

While the existence of interactions among viruses is undisputed, their impact on host susceptibility, transmission, and virulence is unclear, mainly because different clinical [9, 10] and experimental studies [11, 12] have yielded dissimilar—and often contradictory—results. Experimental approaches can provide insight on how intrinsic and extrinsic factors can affect virus-virus interactions. Intrinsic factors could be associated to the virus (eg, virus species and sequence) or the host (eg, immunocompetence and comorbidities). Extrinsic factors could include infectious dose and time elapsed between primary and secondary viral infection.

IAV and RSV circulate during the winter in temperate climates causing significant disease burden. The circulation patterns of SARS-CoV-2 are not yet clear. To characterize interactions between SARS-CoV-2 and IAV and SARS-CoV-2 and RSV within the human respiratory tract, we used a model of ALI cultures of human bronchial epithelium and examined changes between single infections and coinfections in virus replication kinetics, virus spread, and virus-induced lesions.

## METHODS

### Viruses and Cells

RSV strain A2 (American Type Culture Collection, VR-1540), SARS-CoV-2 strain BetaCoV/England/02/2020/EPI_ISL_407073, and IAV H3N2 (A/Norway/3275/2018, provided by the World Influenza Centre) were used. Human bronchial epithelium cells (HBECs) were cultured as described [3]. Cultures were infected with 10^4^ infectious units of each virus as described [3]. For BX795 experiments, ALI cultures were transferred to Pneumacult-ALI medium containing 6 μMol BX795 (or dimethyl sulfoxide) 18 hours before infection.

### Virus Titrations

Vero 6F5 cells (2.5 × 10^5^/mL) were seeded in 48-well plates. Approximately 24 hours later, virus samples were serially diluted in serum-free DMEM and 50 µL of each dilution was added to each well and incubated at 37°C (1 hour) and overlayed with 200 µL of serum-free DMEM, 2% fetal bovine serum, 1% non-essential amino acids, and 1% Avicel. After 40–48 hours, plates were fixed with 8% formaldehyde, permeabilized using 1% Triton-X for 10 minutes and blocked for 30 minutes with 3% bovine serum albumin. For SARS-CoV-2 and SARS-CoV-2/IAV infections, anti-SARS-CoV-2 antibodies were added. For SARS-CoV-2/RSV infections, anti-SARS-CoV-2 and anti-RSV antibodies were combined. For SARS-CoV-2 immunostaining a sheep polyclonal anti-SARS-CoV-2 N antibody (1:1000) [13] in blocking buffer was incubated (1 hour) at room temperature (RT). Cells were washed and a rabbit anti-sheep IgG (Alexa Fluor 555, 1:1000; Abcam ab150182) was incubated at RT for 1 hour. For RSV staining, anti-RSV nucleoprotein (NP; 1:1000; Abcam 22501) was incubated (1 hour) at RT. After washing, rabbit anti-Mouse IgG (Alexafluor 488, 1:1000; Sigma-Aldrich SAB4600056) was incubated (1 hour) at RT. Cells were washed and foci were counted using an EVOSM5000 microscope. Titers below the limit of detection were given the arbitrary value of 1 to represent the values on a logarithmic scale.

To titrate IAV, 3 × 10^5^ MDCK-SIAT cells/mL were seeded in 12-well plates and serial dilutions of virus samples were prepared in serum-free DMEM. Dilutions were added to each well and incubated at 37°C for 1 hour, after which 1 mL of overlay media (2× MEM; [Life technologies 11935046], 1.2% Avicel [FMC BioPolymer], trypsin, treated with N-tosyl-L-phenylalanine chloromethyl ketone (TPCK) 2 μg/mL [Sigma T4376]) was added. Plates were incubated at 37°C and fixed with 8% formaldehyde approximately 72 hours postinfection (hpi). Titers below the limit of detection were given the arbitrary value of 1 to represent the values on a logarithmic scale.

### Hematoxylin and Eosin Staining and Immunostaining

Transwells were fixed in 8% formaldehyde for 16–24 hours, paraffin embedded, sectioned serially, and subject to either hematoxylin and eosin (H&E) staining or immunohistochemistry (IHC). For IHC, sections underwent antigen retrieval using citrate buffer (10 mM, pH 6). Endogenous peroxidase was blocked with 3% H_2_O_2_ in phosphate-buffered saline (PBS; 137 mM NaCl, 2.7 mM KCl, 8 mM Na2HPO_4_, 2 mM KH2PO_4_), followed by an incubation in blocking buffer (5% normal goat serum [S200H-500, VWR International] in PBS). Incubations with primary antibodies in blocking buffer (mouse anti-MxA, 1:1000, clone M143 [14]; rabbit anti-IFITM3, 1:750 [Proteintech, 11714-1-AP]; rabbit anti-ISG15, 1:1000 [Proteintech, 15981-1-AP]) were done overnight at 4°C. Sections were washed and incubated with biotinylated secondary antibodies (anti-mouse IgG, 1:500 [Merck, AP181B]; anti rabbit IgG, 1:500 [Stratech Scientific, 711-065-152]) in blocking buffer for 1 hour at RT, and further washed in PBS and incubated with extravidine peroxidase (1:100; Merck, E2886-1ML), 1 hour at RT, and stained using 0.05% 3,3′-diaminobenzidine (Sigma Aldrich, D8001-5G; 0.012% H_2_O_2_ in PBS). Nuclei were counterstained using Mayer's hemalum. Sections were mounted using DPX mounting media (Merck, 06522-100ML). SARS-CoV-2 staining was performed as described [13]. For IAV staining, sections were incubated for 10 minutes with proteinase K (Dako, S3020) and blocked using 3% rabbit serum (Vector Laboratories, S-5000) in PBS. Anti-NP (EVS, Clone 238, 1:1500) was incubated for 1 hour at RT. Rabbit anti-mouse IgG (Alexafluor 488, 1:1000; Sigma-Aldrich SAB4600056) was incubated for 1 hour at RT. Slides were mounted using Vectashield mounting media containing 4′,6-diamidino-2-phenylindole (DAPI; Vector Laboratories, H-2000). RSV viral antigen staining was performed using anti-RSV Fusion (F, 1:500; Abcam, ab94968) and counterstained using rabbit anti-mouse IgG (Alexafluor 488, 1:1000; Sigma Aldrich, SAB4600056). H&E and IHC images were collected using a slide scanner (Leica, Aperio Versa 8). Immunofluorescence images were collected using a Zeiss LSM 710 confocal microscope.

### Image Analyses

Individual nuclei counts were counted from H&E sections (normalized to a length of 300 µm) using the Nuclei Seg plugin of the Halo image analysis platform. IHC staining was quantified using Leica Aperio image analysis software. Pixels were classified as negative, weak, medium, and strong positive, counted, and divided by the total number of pixels.

### Statistical Analyses

Statistical analysis was done with R [15], version 4.0.5. Mann-Whitney *U* tests were used to investigate significant differences between viral titers. Separate tests were carried out at individual timepoints. *P* values <.05 were considered significant. Where indicated, multiple pairwise comparisons were adjusted using Holm’s method.

## RESULTS

### Cytopathic Changes Within the Respiratory Epithelium Induced by Simultaneous Coinfections Are Driven by Either IAV or RSV, Not by SARS-CoV-2

**Figure 1.**
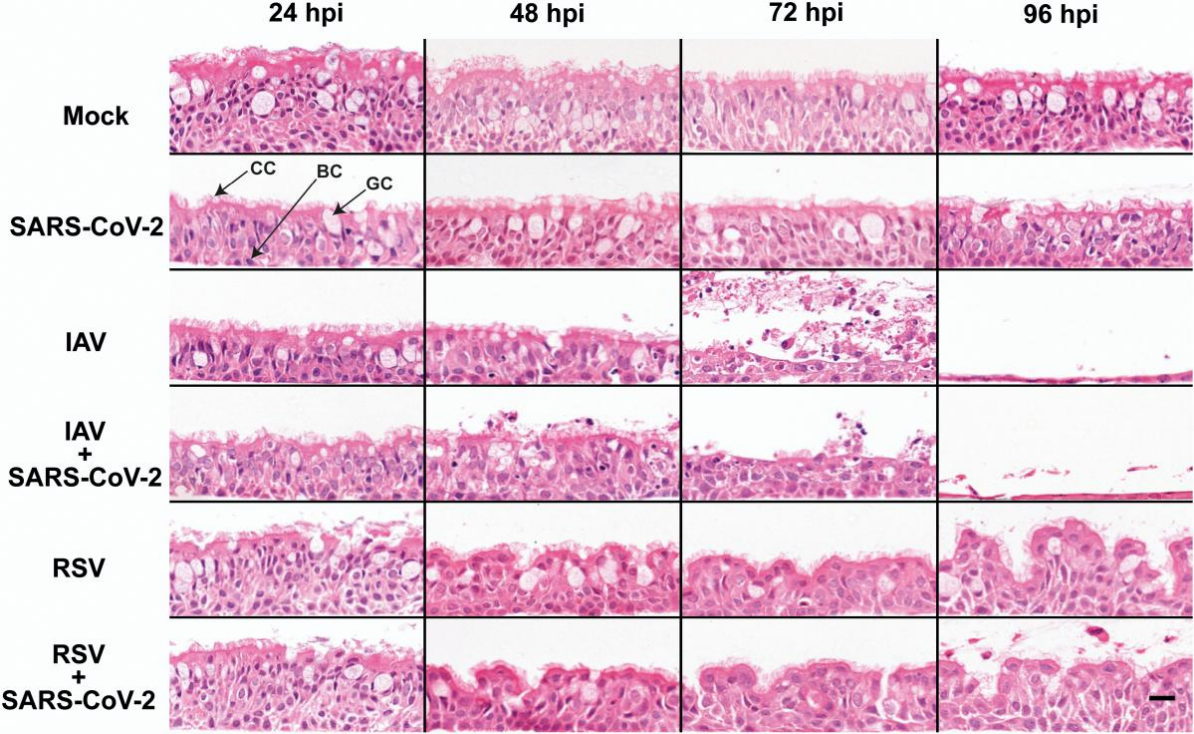
Morphological changes in air-liquid interface cultures of human bronchial cells infected with severe acute respiratory syndrome coronavirus 2 (SARS-CoV-2), influenza A virus (IAV), and respiratory syncytial virus (RSV) in single and coinfections. Representative images of histological sections of cultures infected at different hours postinfection (hpi) with the indicated viruses and stained with hematoxylin and eosin. Images are representative of a minimum of 2 independent experiments. Arrows indicate ciliated cells (CC), basal cells (BC), and goblet cells (GC). Scale bar represents 20 μm.

To determine if SARS-CoV-2 coinfections with either IAV or RSV result in enhanced cytopathology within the respiratory epithelium, we performed single infections (using SARS-CoV-2, IAV, or RSV) and simultaneous coinfections (using SARS-CoV-2/IAV and SARS-CoV-2/RSV) of ALI cultures of HBECs. Cultures were fixed at 24, 48, 72, and 96 hpi, paraffin-embedded, sectioned, and stained with H&E. Epithelial cells were counted, and with the only exception of IAV and IAV/SARS-CoV-2-infected cultures, the number of epithelial cells was relatively stable throughout the duration of our experiments (Supplementary Figure 1). In SARS-CoV-2–infected cultures, the presence and shape of ciliated, basal, and goblet cells seemed unaltered (Figure 1) and no morphological changes were evident compared to the controls. IAV-infected cultures displayed gradual but marked loss of cilia, decreased epithelium thickness, and sloughing of cells (Figure 1). At 72 hpi, there was a significant decrease in the number of cells and by 96 hpi there was an almost complete destruction of the epithelium (Figure 1 and Supplementary Figure 1). RSV-infected cultures acquired a wave-like appearance, which was particularly evident at 96 hpi, but showed no evidence of epithelial thinning (Figure 1). In coinfected cultures, the cytopathic phenotype of IAV/SARS-CoV-2 and RSV/SARS-CoV-2 was indistinguishable from the cultures infected with either IAV or RSV, respectively, suggesting that the presence of SARS-CoV-2 did not enhance or attenuate cytopathogenicity. Overall, our results are consistent with previous reports showing that SARS-CoV-2 does not induce a strong cytopathic effect in the bronchial epithelium [3] and that in coinfections the pattern of lesions is driven by the coinfecting virus.

### SARS-CoV-2 Replication Is Inhibited by Simultaneous Coinfection With IAV or RSV

**Figure 2.**
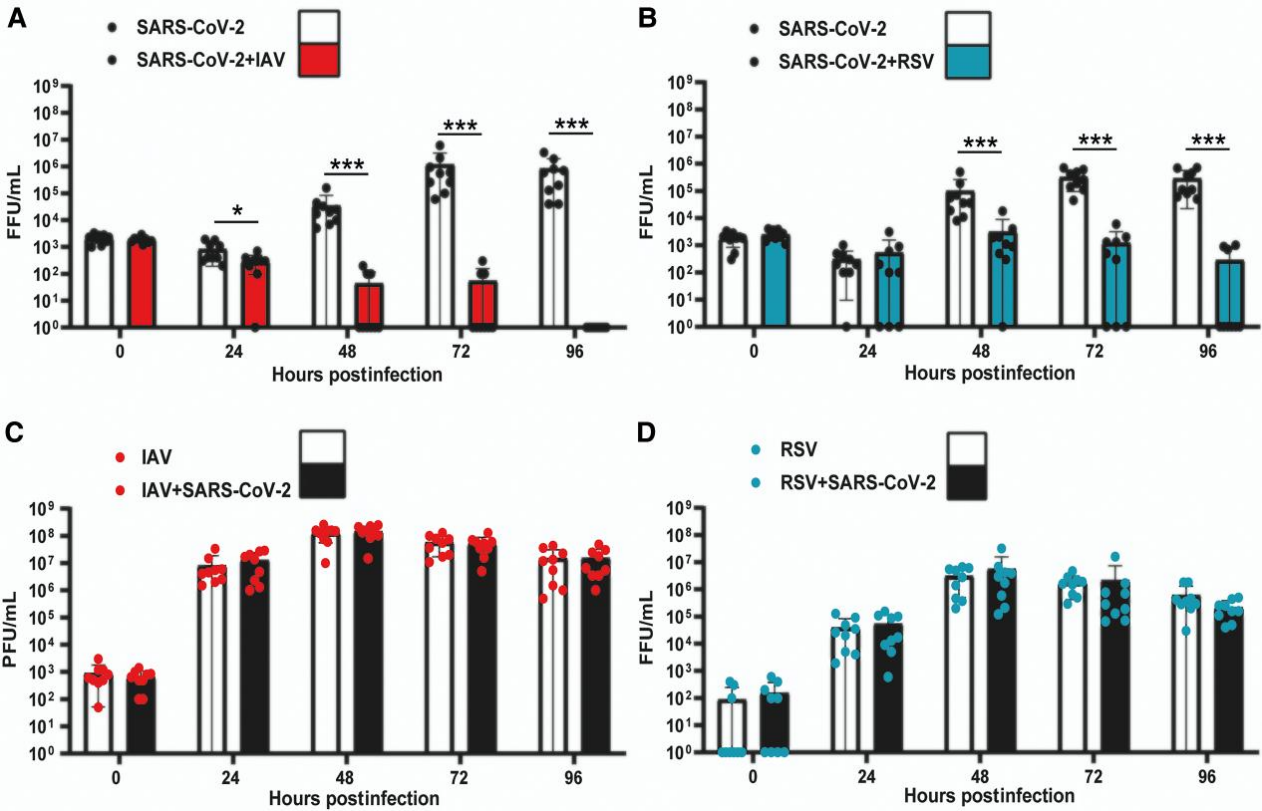
Replication kinetics of SARS-CoV-2, IAV, and RSV in single and coinfections of air-liquid interface cultures of human bronchial cells. *A,* SARS-CoV-2 titers in single SARS-CoV-2 infections (solid black circles, white background) and simultaneous SARS-CoV-2/IAV coinfections (solid black circles, red background). *B,* SARS-CoV-2 titers in single SARS-CoV-2 infections (solid black circles, white background) and simultaneous SARS-CoV-2/RSV coinfections (solid black circles, cyan background). *C,* IAV titers in single IAV infections (solid red circles, white background) and simultaneous SARS-CoV-2/IAV coinfections (solid red circles, background). *D,* RSV titers in single RSV infections (solid cyan circles, white background) and simultaneous SARS-CoV-2/RSV coinfections (solid cyan circles, black ground). Individual titers are shown as circles. Bars represent the mean of 9 values. Error bars represent the standard deviation. Data are combined titers from 3 independent experiments. Statistical significance was tested using Mann-Whitney *U* tests and separate tests were carried out for individual time points. * *P* <.05, *** *P* <.001. Abbreviations: FFU, focus forming units; IAV, influenza A virus; PFU: plaque forming units; RSV, respiratory syncytial virus; SARS-CoV-2, severe acute respiratory syndrome coronavirus 2.

To determine the impact of IAV or RSV on SARS-CoV-2 replication we performed single infections and simultaneous coinfections of ALI cultures of HBECs as described above. We quantified infectious virus from apical washes at 0, 24, 48, 72, and 96 hpi. In single infections, SARS-CoV-2 titers increased gradually after 24 hpi and peaked at 72 hpi (Figure 2*A* and 2*B* ). IAV titers increased at 24 hpi and peaked at 48 hpi (Figure 2*C* ). RSV seemed to replicate at a slower rate than IAV and reached lower peak titers (Figure 2*D* ). In coinfections, SARS-CoV-2 replication was significantly reduced by the presence of either IAV or RSV (Figure 2*A* and 2*B* ). However, the level of reduction differed depending on the coinfecting virus. When SARS-CoV-2 was coinfected with IAV, the observed reduction in SARS-CoV-2 titers was significantly different from 24 hpi onwards (*P* values <.05, Mann-Whitney test). The impact of IAV upon SARS-CoV-2 replication was striking at 48, 72, and 96 hpi. For example, 6/9 coinfected cultures exhibited SARS-CoV-2 titers below the limit of detection at 48 and 72 hpi and by 96 hpi no infectious virus was detectable in any of the infected transwells (n = 9). The replication kinetics of IAV were unaffected by the presence of SARS-CoV-2 (Figure 2*C* ). In coinfections with RSV, SARS-CoV-2 replication was reduced (Figure 2*B* ) and significantly less SARS-CoV-2 infectious virus was detected at 48, 72, and 96 hpi (*P* values <.05, Mann-Whitney test). Notably, the reduction induced by RSV on SARS-CoV-2 replication appeared to not be as strong as that induced by IAV. For example, infectious SARS-CoV-2 was still detectable in 3/9 coinfected cultures at 96 hpi. RSV replication kinetics were also unaffected by SARS-CoV-2 (Figure 2*D* ). Overall, these results show that SARS-CoV-2 replication is severely reduced in the presence of IAV or RSV and that the inhibitory phenotype might be virus specific.

### SARS-CoV-2 Spread Is Reduced in Coinfected Tissues

**Figure 3.**
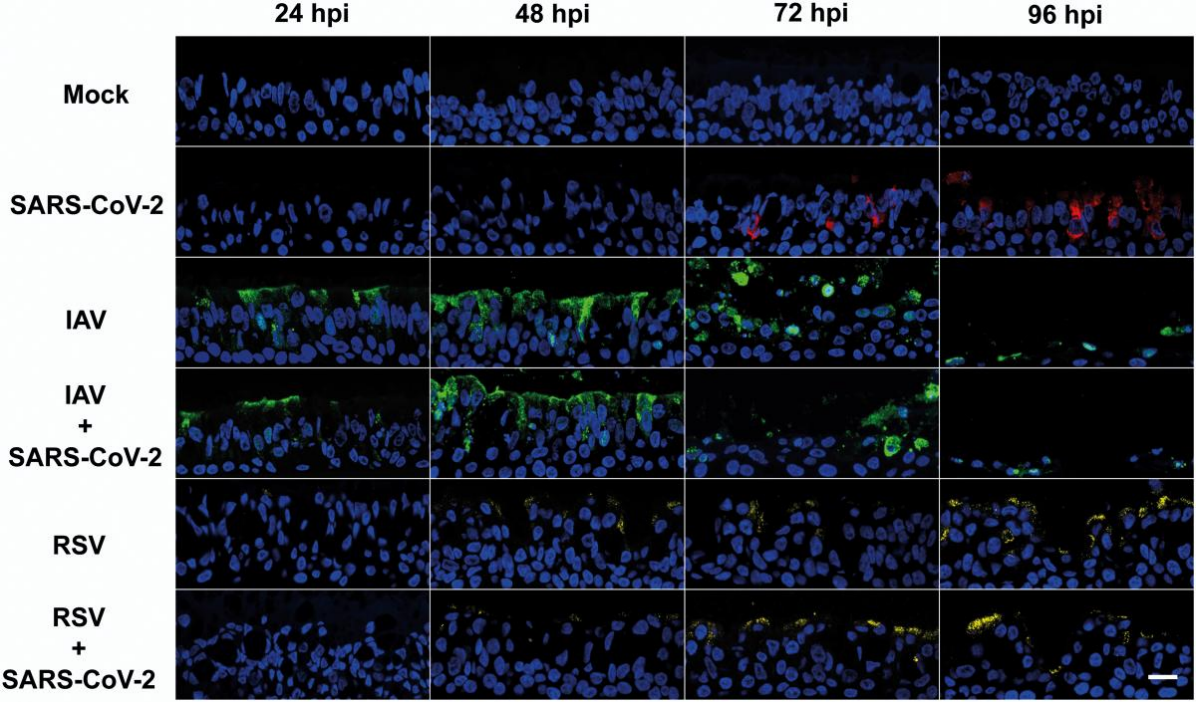
Virus staining in air-liquid interface cultures of human bronchial cells infected with severe acute respiratory syndrome coronavirus 2 (SARS-CoV-2), influenza A virus (IAV), and respiratory syncytial virus (RSV) in single and coinfections. Representative images of histological sections of cultures infected at different hours postinfection (hpi) with the indicated viruses and stained with antibodies against SARS-CoV-2 nucleocapsid (red), IAV nucleoprotein (green), and RSV fusion protein (yellow). Nuclei were stained using 4′,6-diamidino-2-phenylindole (DAPI). Images are representative of a minimum of 2 independent experiments. Scale bar represents 20 μm.

We hypothesized that SARS-CoV-2 spread within the respiratory epithelium would be reduced. To test this hypothesis, we performed immunofluorescence with antibodies against SARS-CoV-2 nucleocapsid protein (N), IAV nucleoprotein (NP), and RSV fusion protein (F) to sections of the same cultures used in Figure 1. Figure 3 shows that in single infections, SARS-CoV-2–positive cells were detectable at 72 hpi and fluorescence signal increased at later time points, mainly on the apical portion of the epithelium. In contrast, IAV-positive cells were readily observed at 24 hpi and by 48 hpi most of the apical epithelium displayed IAV-NP antigen staining, including sloughed cells (Figure 1). At 96 hpi, IAV staining decreased drastically but this was likely due to a reduction on epithelial cells as a result of virus-induced cytopathology. RSV signal was detected after 24 hpi (Figure 3). For each individual virus infection, the relative timings for viral antigen positivity were consistent with the observed peaks in replication kinetics. In coinfections, we rarely observed SARS-CoV-2–positive cells, whereas staining patterns for IAV and RSV were similar to those observed in single and coinfections. Overall, these results indicate that SARS-CoV-2 spread across the respiratory epithelium is drastically reduced by coinfection with IAV or RSV and this reduction is consistent with the observed decrease in SARS-CoV-2 replication.

### IAV and RSV Induce Stronger Innate Immune Responses Than SARS-CoV-2 in the Bronchial Epithelium

**Figure 4.**
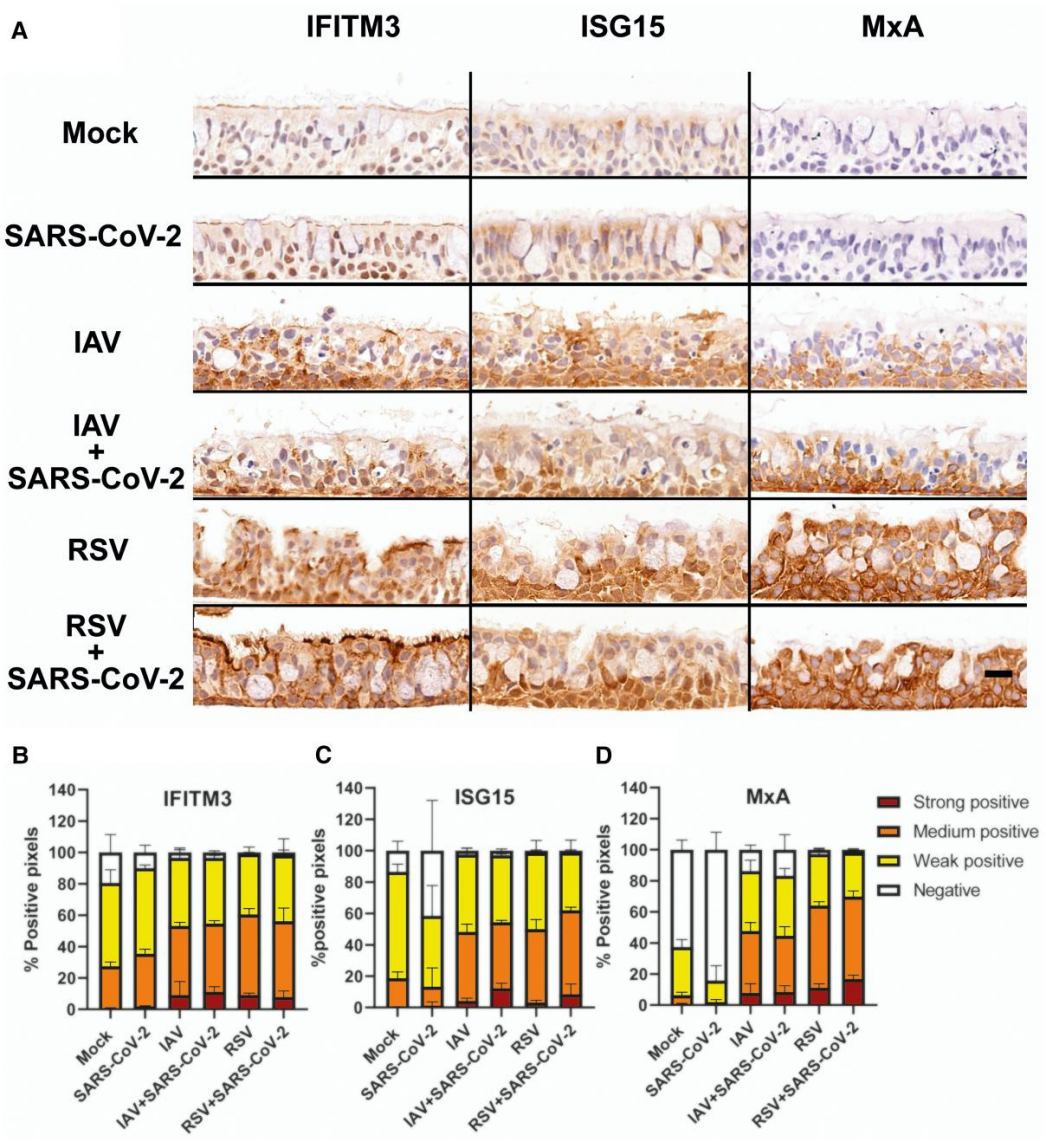
Expression of interferon-stimulated genes in air-liquid interface cultures of human bronchial cells infected with severe acute respiratory syndrome coronavirus 2 (SARS-CoV-2), influenza A virus (IAV), and respiratory syncytial virus (RSV) in single and coinfections. *A,* Representative light microscopy images of IFITM3, ISG15, and MxA expression detected by immunohistochemistry at 48 hours postinfection. Air-liquid interface cultures were mock infected, infected with SARS-CoV-2 only, IAV only, RSV only, and coinfected with SARS-CoV-2 and IAV or with SARS-CoV-2 and RSV. Positive immunostaining is colored brown. Scale bar represents 20 μm. *B*, *C,* and *D*, Bar plots showing quantification of staining signal by IFITM3, ISG15, and MxA, respectively.

To compare the timing and extent of the innate immune response triggered by either SARS-CoV-2, IAV, or RSV in single and coinfections in the bronchial epithelium, we examined the expression levels of 3 interferon-stimulated genes (ISGs) with known antiviral activities: myxovirus resistance protein A (MxA) [3, 13, 16], interferon-induced transmembrane protein 3 (IFITM3) [17], and ISG15 [18]. Figure 4 shows images of Formalin-fixed, paraffin-embedded (FFPE) sections of cultures infected with either SARS-CoV-2, IAV, or RSV, or coinfected simultaneously with SARS-CoV-2/IAV or with SARS-CoV-2/RSV at 48 hpi. This time point was selected because all conditions exhibited a similar number of cells (Supplementary Figure 1). As the 3 ISGs examined are constitutively expressed, we measured their relative intensity. Levels of protein expression were classified as weak, medium, or strong (see “Methods”). Cultures infected with SARS-CoV-2 exhibited the lowest expression of all 3 ISGs when compared with those infected by IAV or RSV, or coinfected (Figure 4). Indeed, detection of strong staining for any ISG was virtually absent in cultures infected with SARS-CoV-2 only (Figure 4). Supplementary Figures 2, 3, and 4 show IHC staining patterns from 24 to 96 hpi for IFITM3, ISG15, and MxA, respectively. These results suggest that SARS-CoV-2 triggers a weaker innate immune response compared to IAV and RSV but in simultaneous coinfections the response against IAV and RSV is dominant.

### Impairment of the Innate Immune Response Rescues SARS-CoV-2 Replication in Coinfected Tissues

**Figure 5.**
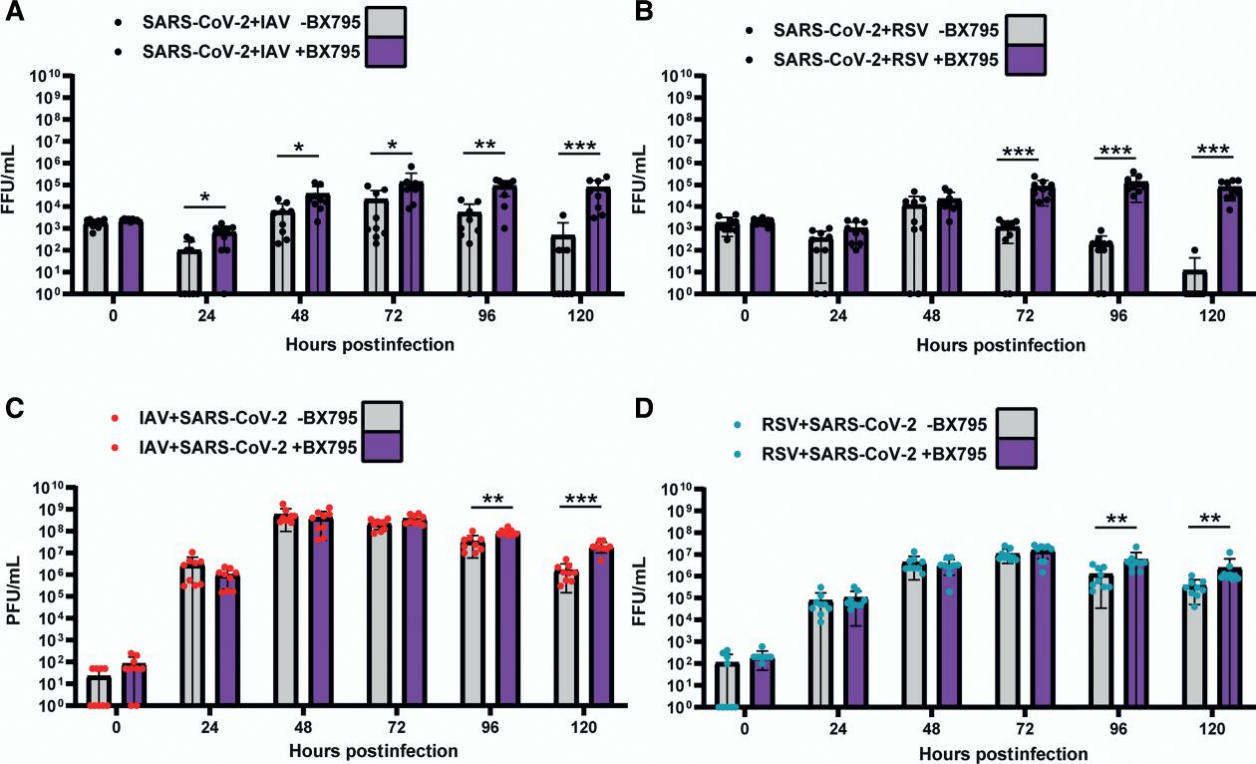
Replication kinetics of SARS-CoV-2, IAV, and RSV in air-liquid interface cultures of human bronchial cells coinfected simultaneously with SARS-CoV-2 and IAV or SARS-COV-2 and RSV in the presence (purple bars) or absence of BX795 (grey bars). *A*, SARS-CoV-2 titers in coinfections with IAV. *B*, SARS-CoV-2 titers in coinfections with RSV. *C*, IAV titers in coinfections with SARS-CoV-2. *D*, RSV titers in coinfections with SARS-CoV-2. SARS-CoV-2 titers are shown in black, IAV in red, and RSV in cyan. Individual titers are shown as circles. Bars represent the mean of 9 values. Error bars represent the standard deviation. Data are combined titers from 3 independent experiments. Statistical significance was tested using Mann-Whitney *U* tests and separate tests were carried out for individual time points. * *P* <.05, ** *P* <.01, *** *P* <.001. Abbreviations: FFU, focus forming units; IAV, influenza A virus; PFU: plaque forming units; RSV, respiratory syncytial virus; SARS-CoV-2, severe acute respiratory syndrome coronavirus 2.

**Figure 6.**
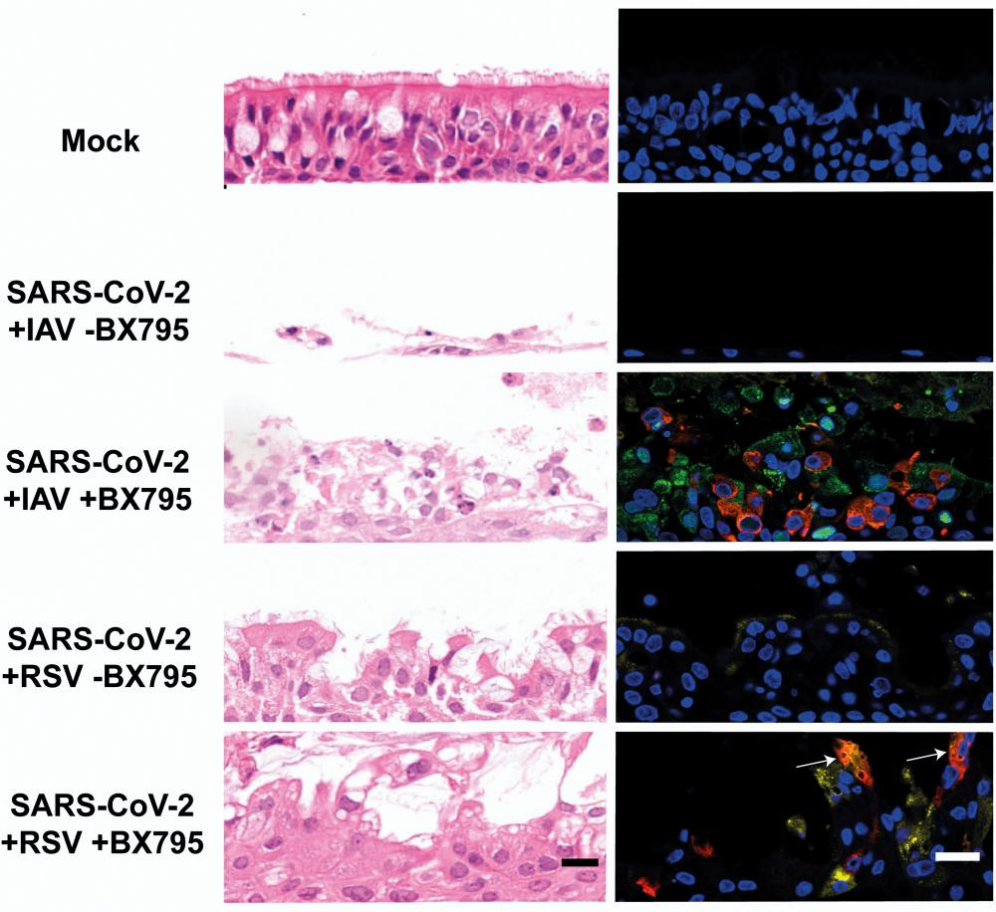
Morphological changes and virus spread in air-liquid interface cultures of human bronchial cells coinfected with severe acute respiratory syndrome coronavirus 2 (SARS-CoV-2) and influenza A virus (IAV) or SARS-CoV-2 and respiratory syncytial virus (RSV) in the presence or absence of BX795. Left column shows representative images of histological sections stained with hematoxylin and eosin of cultures coinfected with the indicated viruses at 120 hours postinfection with the indicated viruses. Right column shows representative immunofluorescence images of histological sections stained with antibodies against SARS-CoV-2 nucleocapsid (red), IAV nucleoprotein (green), and RSV fusion protein (yellow). Nuclei were stained using 4′,6-diamidino-2-phenylindole (DAPI). White arrows indicate cells positive for both SARS-CoV-2 and RSV antigens. Images are representative of 3 independent experiments. Scale bars represent 20 μm.

To determine if the block to SARS-CoV-2 replication was due to innate immune responses triggered by IAV or RSV, we performed simultaneous coinfections of ALI HBECs in the presence or absence of BX795, a drug that impairs the type I IFN response by inhibiting the phosphorylation of IRF-3 [2]. Figure 5*A* shows that in coinfections with IAV in the presence of BX795, SARS-CoV-2 replicated to significantly (*P* values <.05, Mann-Whitney test) higher levels than in the controls. Figure 5*B* shows that BX795 also increased the titers of SARS-CoV-2 in coinfections with RSV. This was particularly evident from 72 hpi onwards, when differences in viral titers were statistically significant (*P* values <.05, Mann-Whitney test). Both IAV and RSV reached marginally but significantly (*P* values <.05, Mann-Whitney test) higher titers in the presence of BX795 (Figure 5*C* and 5*D* ). BX795 did not affect the overall replication of SARS-CoV-2 in single infections (Supplementary Figure 5) as a statistically significant increase in SARS-CoV-2 titer was observed only at 120 hpi. To determine morphological changes in respiratory epithelia supporting replication of SARS-CoV-2 and either IAV or RSV we examined H&E sections of FFPE HBECs that had been coinfected in the presence or absence of BX795. Coinfection with SARS-CoV-2 and IAV in the presence of BX795 caused a lower degree of lesions in the epithelium when compared to coinfections in the absence of the drug (Figure 6), which could be due to a reduction in apoptosis triggered by the innate immune response [19]. This was not the case in coinfections with SARS-CoV-2 and RSV. We performed immunostaining for viral antigens as described above and, in contrast to untreated cultures, in BX795-treated cultures viral antigens for both viruses (either SARS-CoV-2 and IAV or SARS-CoV-2 and RSV) were observed in coinfections (Figure 6). Coinfected cultures displayed a mixture of single and coinfected cells, suggesting that interactions between the viruses did not result in SARS-CoV-2 exclusion. These results show that (1) the block in SARS-CoV-2 replication in coinfections is due to the innate immune response triggered by IAV or RSV; and (2) that cellular coinfections can occur in the absence of an innate immune response.

### Relative Timings of Infections Affect SARS-CoV-2 Susceptibility to Inhibition by Superinfecting Viruses

To test if the time elapsed between primary and secondary viral infections had an effect on SARS-CoV-2 replication, we infected ALI cultures of HBECs with SARS-CoV-2 (set as time zero) and superinfected them at different timepoints with either IAV or RSV (Supplementary Figure 6*A* and 6*C*, respectively). Supplementary Figure 6*B* shows that the biggest reduction in SARS-CoV-2 titers was observed when IAV was added 24 hours after SARS-CoV-2 infection. Specifically, SARS-CoV-2 replicated at similar levels in the presence or absence of IAV up to 48 hpi (Supplementary Figure 6*B*), after which SARS-CoV-2 titers were significantly lower, apart from 120 hpi (*P* values <.05, Mann-Whitney test) in cultures where IAV was present. When IAV was inoculated at 72 hours after SARS-CoV-2 infection, SARS-CoV-2 replication was similar to that observed in mock-superinfected controls. SARS-CoV-2 exhibited significantly lower titers (*P* values <.05, Mann-Whitney test) than the controls at 168 and 192 hpi (Supplementary Figure 6*B*). Supplementary Figure 6*D* shows staggered infections using SARS-CoV-2 and RSV. A significant level of inhibition was seen on SARS-CoV-2 replication when infected cultures were challenged with RSV 24 hours after SARS-CoV-2 infection as 5/9, 8/9, and 9/9 of infected cultures did not have detectable SARS-CoV-2 at 144, 168, and 192 hpi, respectively. In cultures challenged with RSV 72 hours after SARS-CoV-2 infection, differences in SARS-CoV-2 titers were nonsignificant when compared to mock-challenged controls (Supplementary Figure 6*D*), albeit at 192 hpi 4/9 infected cultures did have detectable levels of SARS-CoV-2 compared to 7/9 cultures for the mock challenged. These experiments showed that the shorter the time between infections, the stronger the block in SARS-CoV-2 replication regardless of the superinfecting virus.

## DISCUSSION

Virus-virus interactions impact the infection dynamics of respiratory viruses [1]. Nonpharmaceutical interventions aiming at reducing the transmission of SARS-CoV-2 also reduced the incidence of other respiratory viruses [20–23]. However, as restrictions were lifted in many countries in 2022, it is likely that the incidence of viral coinfections will increase. It is therefore essential to understand better the biology of SARS-CoV-2 coinfections at the within-host scale as this will affect viral pathogenesis and transmission. Using a coinfection model of airway epithelium, we characterized interactions between SARS-CoV-2, IAV, and RSV. HBECs are routinely used to study the infection biology of respiratory viruses because they recapitulate to a large degree the natural site of infection [24–27]. We performed single infections and coinfections and examined replication kinetics, virus spread within the epithelium, histopathological changes, and innate immune activation.

SARS-CoV-2 is susceptible to IFN [28] and our results show that IAV and RSV block SARS-CoV-2 replication by triggering an antiviral response. Previous studies showed that HRV also generates an antiviral response that blocks SARS-CoV-2 [3, 29]. This 3-way interaction whereby a response against infection with a primary virus interferes with a secondary virus has been described using ALI cultures of respiratory epithelium for other virus combinations, including HRV and IAV [2] as well as RSV and HRV [4]. Considering the results presented here and the work published by other groups, it is reasonable to propose that virus-induced IFN-mediated interference is a major contributor of within-host negative interactions. However, other factors, such as reduction of viable cells due to virus-induced cell death might also contribute to the interfering phenotype. We showed that the degree of interference is likely to be virus-specific as IAV seems to block SARS-CoV-2 more efficiently than RSV. It is possible that the magnitude of interference will depend on the potency of the IFN response elicited by the primary virus and the susceptibility to the IFN response of the secondary virus, albeit in the case of IAV, the increased cell death observed from 72 hpi onwards may have acted as an additional factor. Transcriptomics-based studies coupled with IFN-stimulated genes screens [30] might pinpoint specific genes or intracellular pathways that explain such differences. Similarly, coinfection studies using SARS-CoV-2 variants will be important to assess how SARS-CoV-2 adaptation impacts viral interference, as evolution affects the ability of the virus to overcome the IFN response [31]. Another variable that affects the potency of interference is the time between primary and secondary infections. This is important as it would be expected that simultaneous coinfections would be less frequent than superinfections.

The reduced expression of IFITM3, ISG15, and MxA in SARS-CoV-2 single-infected cultures highlighted the weak immune response generated by this virus and contrasted with the broad innate immune activation triggered by IAV or RSV. However, these results should be interpreted with caution as systemic responses are important contributors to pathogenesis, and they are absent in ALI cultures.

Studying interactions among respiratory viruses is critical: approximately 10% of viral respiratory infections are coinfections [32], and most coinfections affect children <5 years old. Studying coinfections experimentally is challenging due to the combination of factors that can affect coinfection phenotypes, including the use of different cell types (ie, nasal, tracheal, or bronchial), incubation temperatures (lower temperatures can be used to mimic the upper respiratory environment whereas higher temperatures would mimic hyperthermia), virus strains, and inoculum doses used. A previous coinfection study using SARS-CoV-2, IAV, and RSV in ALI cultures of nasal cells found that IAV but not RSV could reduce SARS-CoV-2 replication and that RSV replication was significantly affected by coinfecting SARS-CoV-2 [33]. Such challenges also apply to *in vivo* studies using animal models. For example, a mouse model study showed that coinfection with IAV and HRV caused milder influenza but did not reduce IAV shedding [8], whereas coinfection with IAV and mouse hepatitis virus strain 1, a murine coronavirus, attenuated disease presentation and reduced IAV replication [7, 8], and this was associated with IFN upregulation. In contrast, other studies that investigated *in vivo* coinfections of IAV and SARS-CoV-2 showed that disease severity was increased in coinfections [34–36] and SARS-CoV-2 replication was reduced [34, 37]. Similar results were also observed in patients that were positive for both viruses [38, 39].

In summary, our results, together with those reported by previous studies, suggest that SARS-CoV-2 is particularly susceptible to negative interference by other respiratory viruses that trigger a strong IFN response. If this effect is translated at the epidemiological scale, it is feasible to speculate that the incidence of SARS-CoV-2 infections will decrease in future winter seasons as normal mixing resumes.

## Supplementary Data

Supplementary materials are available at *The Journal of Infectious Diseases* online. Consisting of data provided by the authors to benefit the reader, the posted materials are not copyedited and are the sole responsibility of the authors, so questions or comments should be addressed to the corresponding author.

## Supplementary Material

jiac494_Supplementary_Data

## Notes

***Acknowledgment.*** We thank Lynn Stevenson, Frazer Bell, and Lynn Oxford for technical assistance.

***Financial support.*** This work was supported by the Medical Research Council of the United Kingdom (grant number MC_UU_12014/9).
