# Supplementary figures and images for: Influenza A and Respiratory Syncytial Virus Trigger a Cellular Response That Blocks Severe Acute Respiratory Syndrome Virus 2 Infection in the Respiratory Tract
Source: J Infect Dis. 2022 Dec 23;227(12):1396–406. doi: 10.1093/infdis/jiac494 (PMC10266949; doi:10.1093/infdis/jiac494)

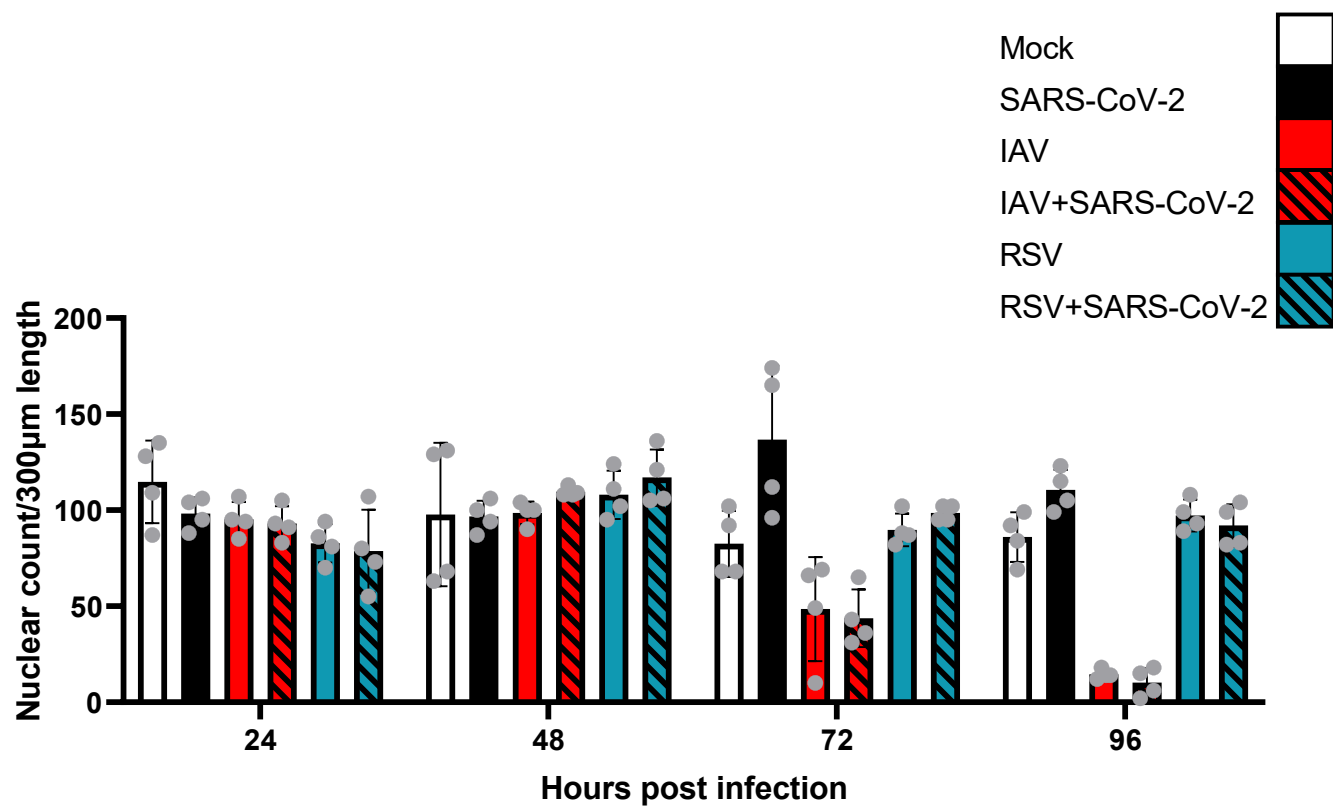

Supplement: jiac494_Supplementary_Data [file jiac494_supplementary_data.zip › Supplementary_figure_1.pdf]

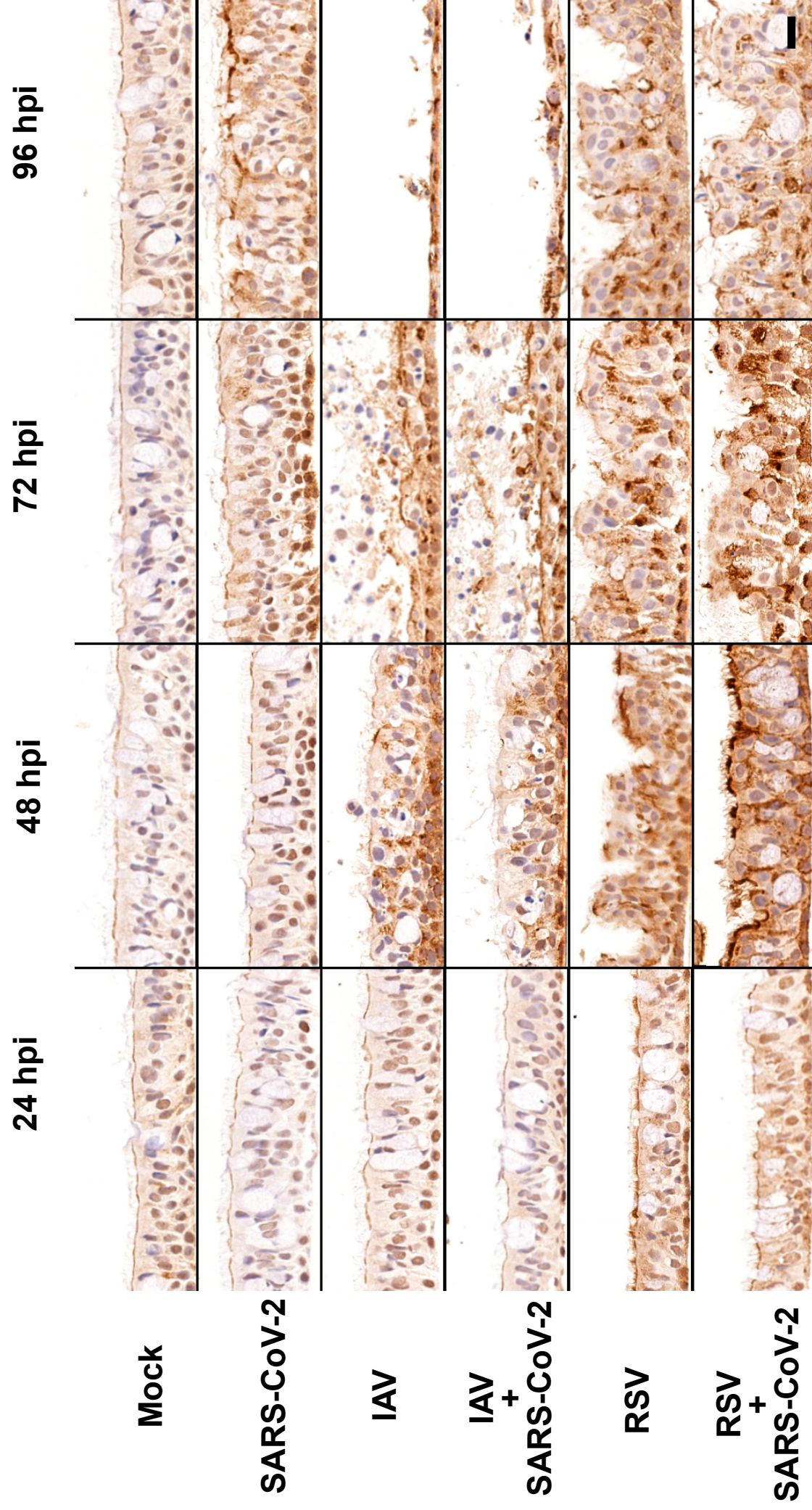

Supplement: jiac494_Supplementary_Data [file jiac494_supplementary_data.zip › Supplementary_figure_2.pdf]

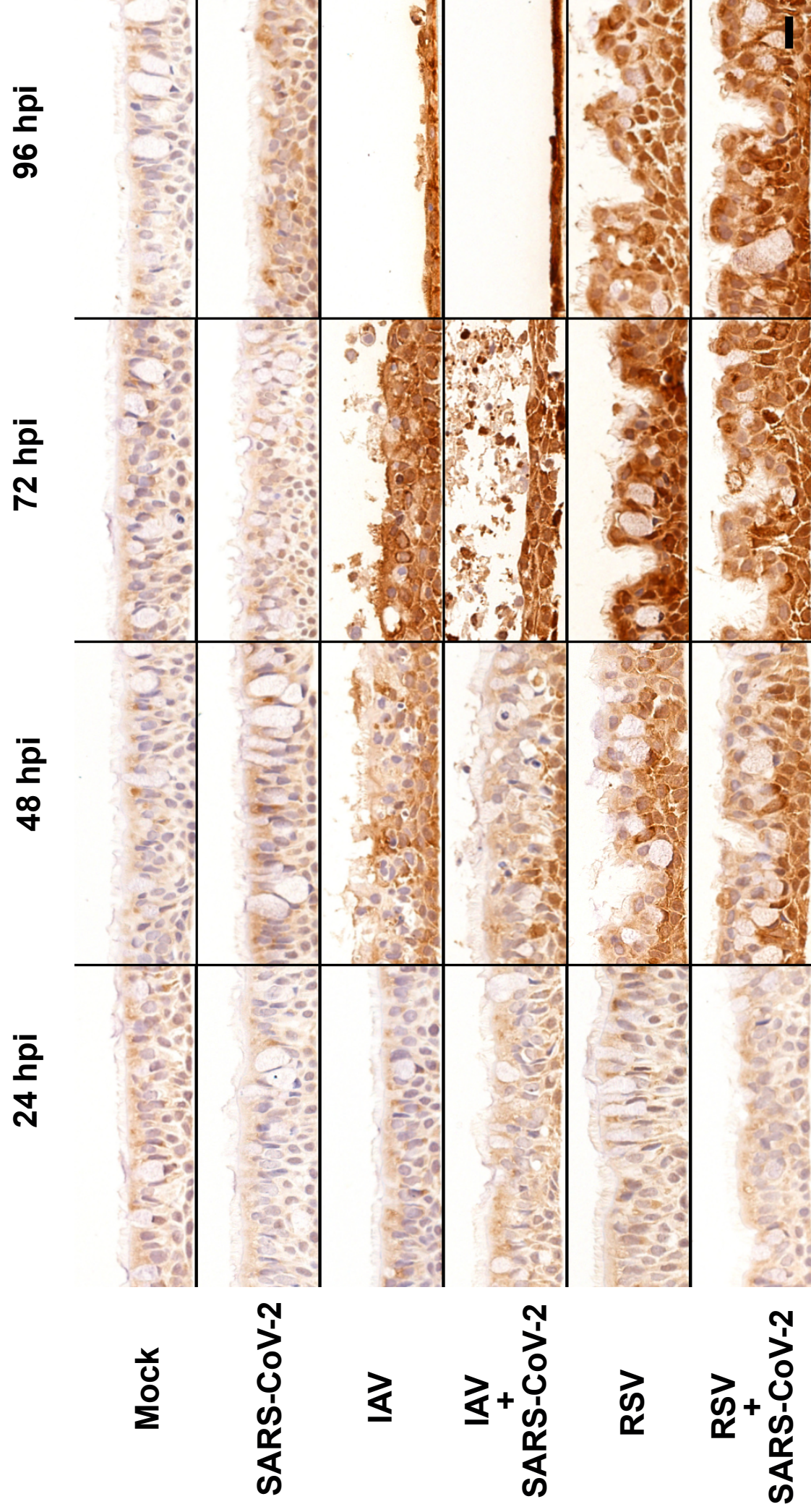

Supplement: jiac494_Supplementary_Data [file jiac494_supplementary_data.zip › Supplementary_figure_3.pdf]

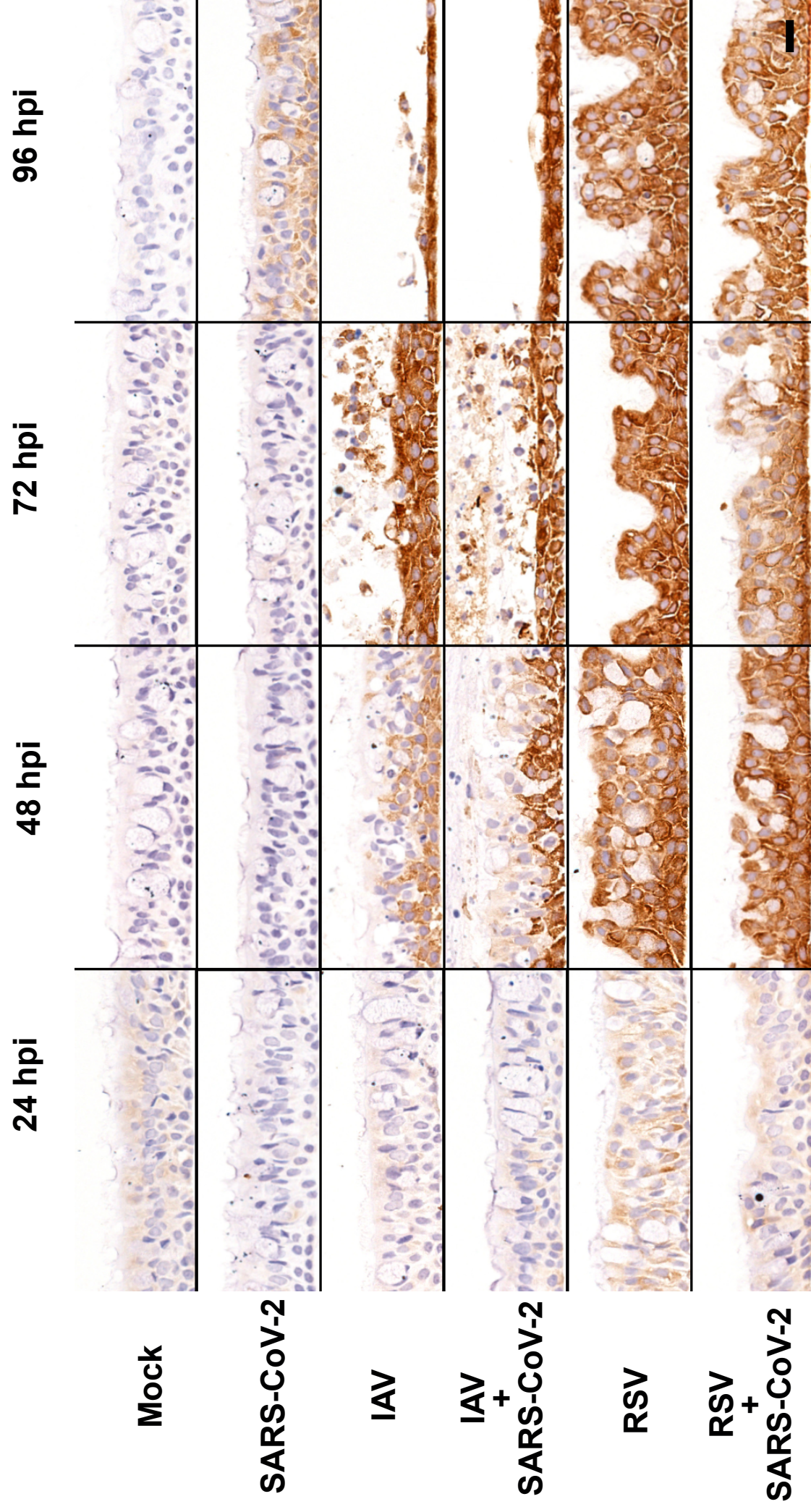

Supplement: jiac494_Supplementary_Data [file jiac494_supplementary_data.zip › Supplementary_figure_4.pdf]

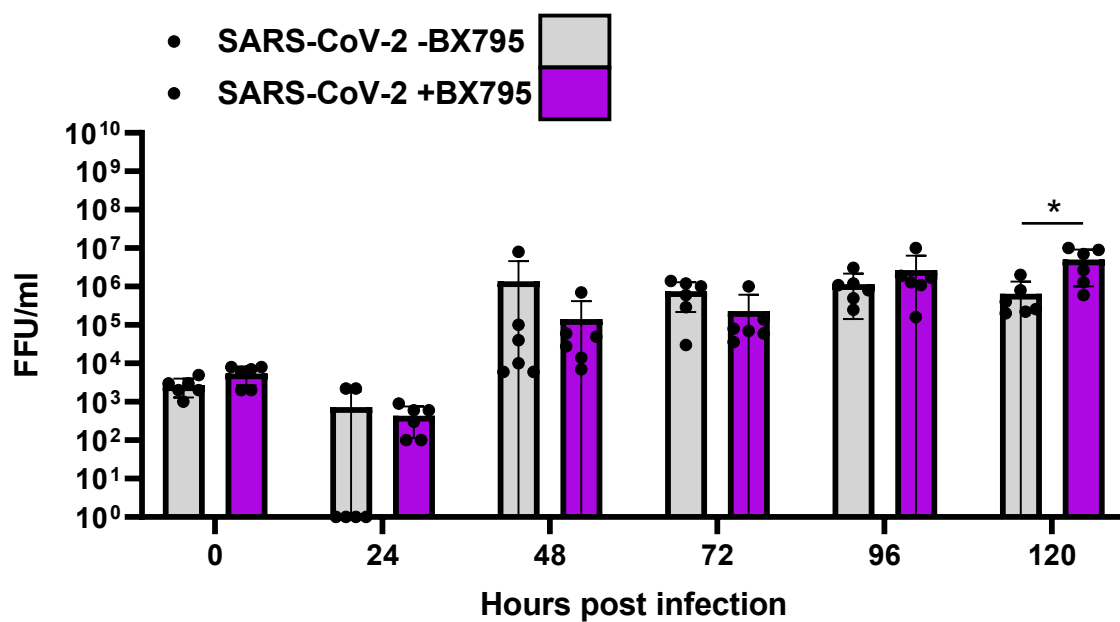

Supplement: jiac494_Supplementary_Data [file jiac494_supplementary_data.zip › Supplementary_figure_5.pdf]

**A**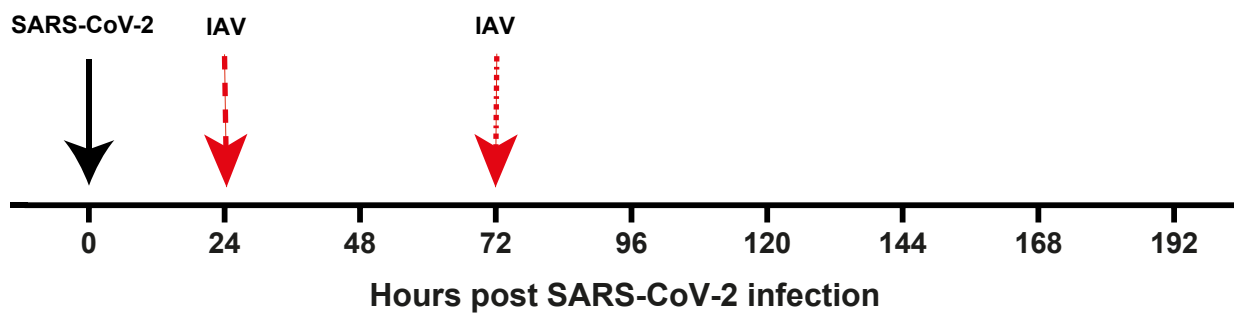**B**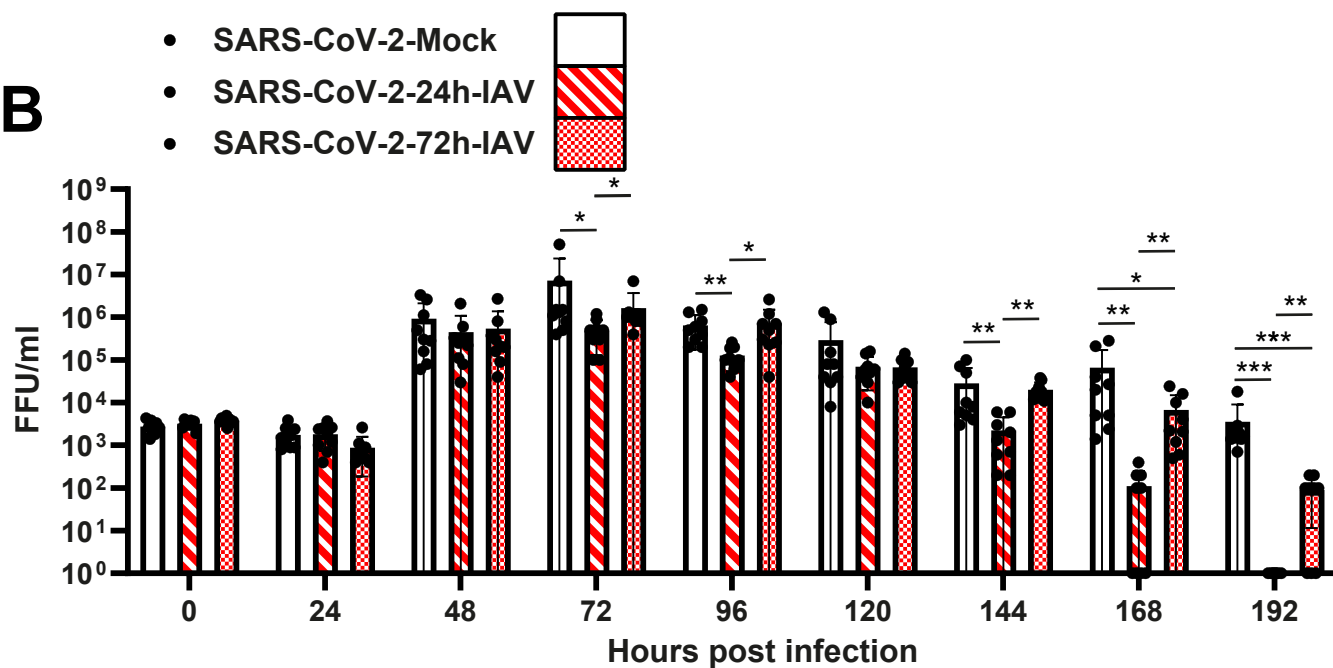**C**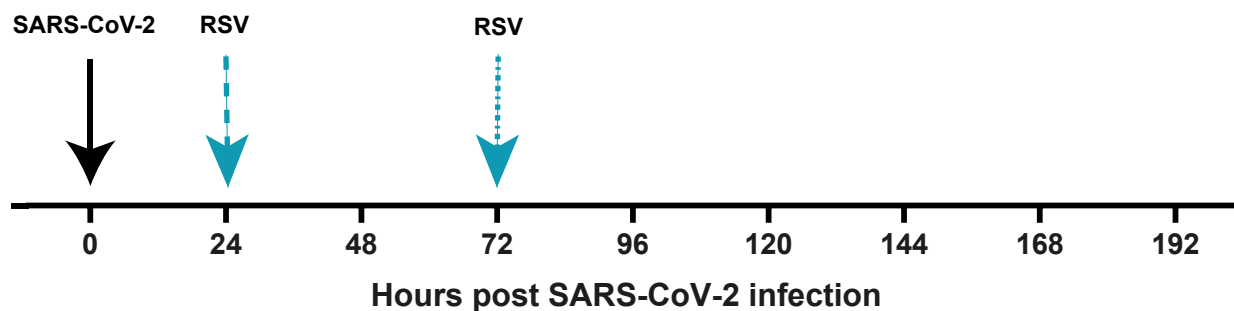**D**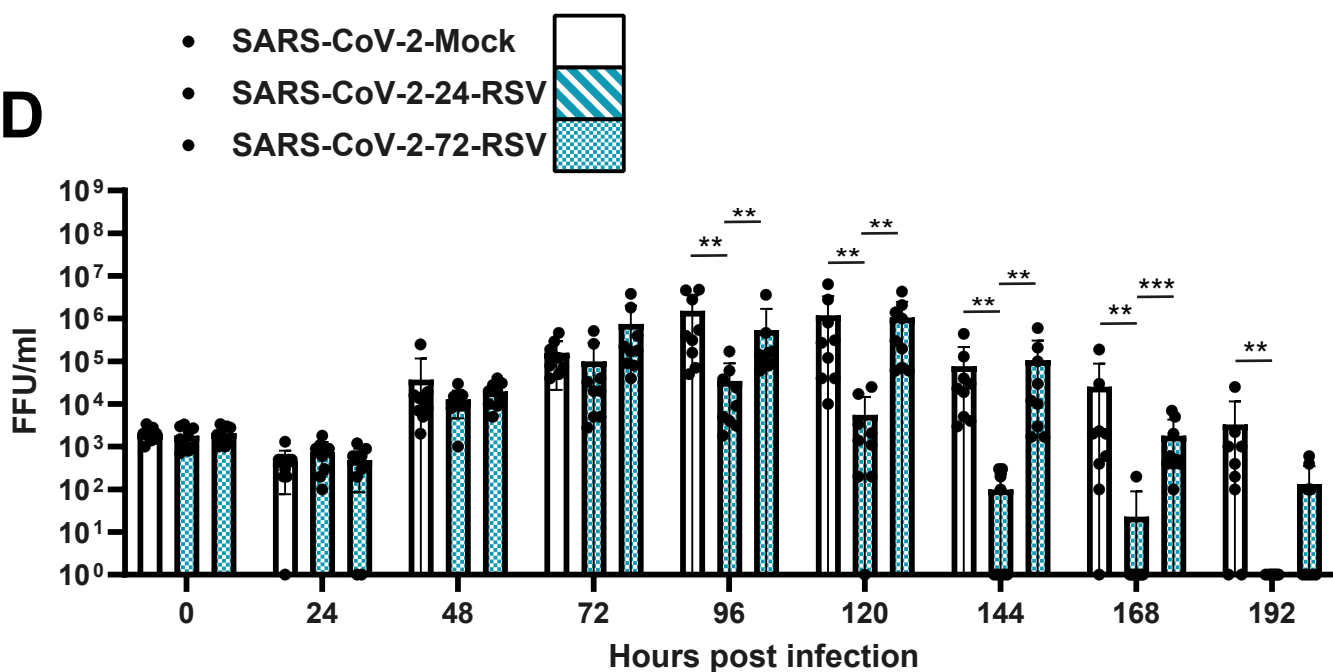

Supplement: jiac494_Supplementary_Data [file jiac494_supplementary_data.zip › Supplementary_figure_6.pdf]
